# Comparison of pre- and postoperative myocardial injuries on mortality after non-cardiac surgery: a retrospective analysis using an inverse probability weighting adjustment

**DOI:** 10.1038/s41598-020-78023-9

**Published:** 2020-12-03

**Authors:** Seung-Hwa Lee, Jungchan Park, Jong-Hwan Lee, Jeong Jin Min, Kwan Young Hong, Hyojin Cho, Keumhee Carriere, Joonghyun Ahn

**Affiliations:** 1 Division of Cardiology, Department of Medicine, Heart Vascular Stroke Institute, Samsung Medical Center, Sungkyunkwan University School of Medicine, Seoul, Korea; 2 Department of Anesthesiology and Pain Medicine, Samsung Medical Center, Sungkyunkwan University School of Medicine, 81 Irwon-ro, Gangnam-gu, Seoul, 06351 Korea; 3 Department of Mathematical and Statistical Sciences, University of Alberta, Edmonton, AB Canada; 4 Statistics and Data Center, Samsung Medical Center, Sungkyunkwan University School of Medicine, Seoul, Korea

**Keywords:** Cardiology, Medical research, Risk factors

## Abstract

Although both pre- and postoperative myocardial injuries are strongly associated with an increased postoperative mortality, no study has directly compared the effects of pre- and postoperative myocardial injuries on 30-day mortality after non-cardiac surgery. Therefore, we evaluated and compared the effects of pre- and postoperative myocardial injury on 30-day mortality after non-cardiac surgery. From January 2010 to December 2016, patients undergoing non-cardiac surgery were stratified into either the normal (*n* = 3182), preoperative myocardial injury (*n* = 694), or postoperative myocardial injury (*n* = 756) groups according to the peak cardiac troponin value. Myocardial injury was defined as a sole elevation of cardiac troponin value above the 99th percentile upper reference limit without ischemic symptom using the 4th universal definition of myocardial infarction. Patients in the preoperative myocardial injury group were further divided into the attenuated (*n* = 177) or persistent myocardial injury group (*n* = 517) according to the normalization of cardiac troponin level in postoperative period. As the primary outcome, postoperative 30-day mortalities were compared among the groups using the weighted Cox proportional-hazards regression models with the inverse probability weighting. Compared with the normal group, postoperative 30-day mortality was increased significantly both in the pre- and postoperative myocardial injury groups (1.4% vs*.* 10.7%; hazard ratio [HR] 3.12; 95% confidence interval [CI] 1.62–6.01; *p* = 0.001 and 1.4% vs*.* 7.4%; HR 4.49; 95% CI 2.34–8.60; *p* < 0.001, respectively), however, there was no difference between the pre- and postoperative myocardial injury groups (HR, 1.44; 95% CI 0.79–2.64; *p* = 0.45). In addition, the attenuated myocardial injury group showed a significantly lower postoperative 30-day mortality than the persistent myocardial injury group (5.6% vs*.* 12.4%; HR 2.23; 95% CI 1.17–4.44; *p* = 0.02). In patients undergoing non-cardiac surgery, preoperative myocardial injury also increased postoperative 30-day mortality to a similar degree of postoperative myocardial injury. Further studies on the importance of preoperative myocardial injury are needed.

**Clinical trial number and registry URL:** KCT0004348 (www.cris.nih.go.kr).

## Introduction

More than 1% of patients aged 45 years or older die during early postoperative period after major non-cardiac surgery^1,2^. This mortality rate is about 1000 times greater than anaesthesia-related intraoperative mortality that has currently decreased to less than 0.001%^3,4^. Myocardial injury and infarction are considered as the leading causes of the postoperative mortality, accounting for a quarter of all postoperative deaths^5^. However, since most postoperative myocardial injury and/or infarction occurs within 2 days after non-cardiac surgery when cardiac symptoms can be masked by analgesic medication^6^, postoperative myocardial injury sometimes can be identified only by the cardiac troponin (cTn) elevation.

Many previous studies have shown that pre- and postoperative cTn elevations, regardless of ischemic signs and/or symptoms, are strongly associated with postoperative mortality in patients undergoing non-cardiac surgery^1,6–13^. In addition, myocardial injury was clearly differentiated from type 2 myocardial infarction based on the presence of signs and/or symptoms of clinical myocardial ischemia and specified into acute and chronic form according to the changes in hs-cTn level in the recently published 4th universal definition of myocardial infarction^14^.

However, to the best of our knowledge, there has been no study to compare the effects of pre- and postoperative myocardial injury on 30-day mortality after non-cardiac surgery directly. In addition, diagnostic criteria and clinical impact of perioperative myocardial injury based on the hs-cTn level changes appear not to be fully established. Considering that significant preoperative cTn elevation is found in more than 13% of patients^6^ and that it is strongly associated with high postoperative mortality^10^, the effect of preoperative myocardial injury on the postoperative deaths in non-cardiac surgery might need to be evaluated and compared, separately form that of postoperative myocardial injury. Therefore, the aim of our study was to evaluate and compare the effects of pre- and postoperative myocardial injuries, defined by the peak hs-cTn level according to the 4th universal definition of myocardial infarction, on 30-day mortality after non-cardiac surgery.

## Results

**Figure 1:**
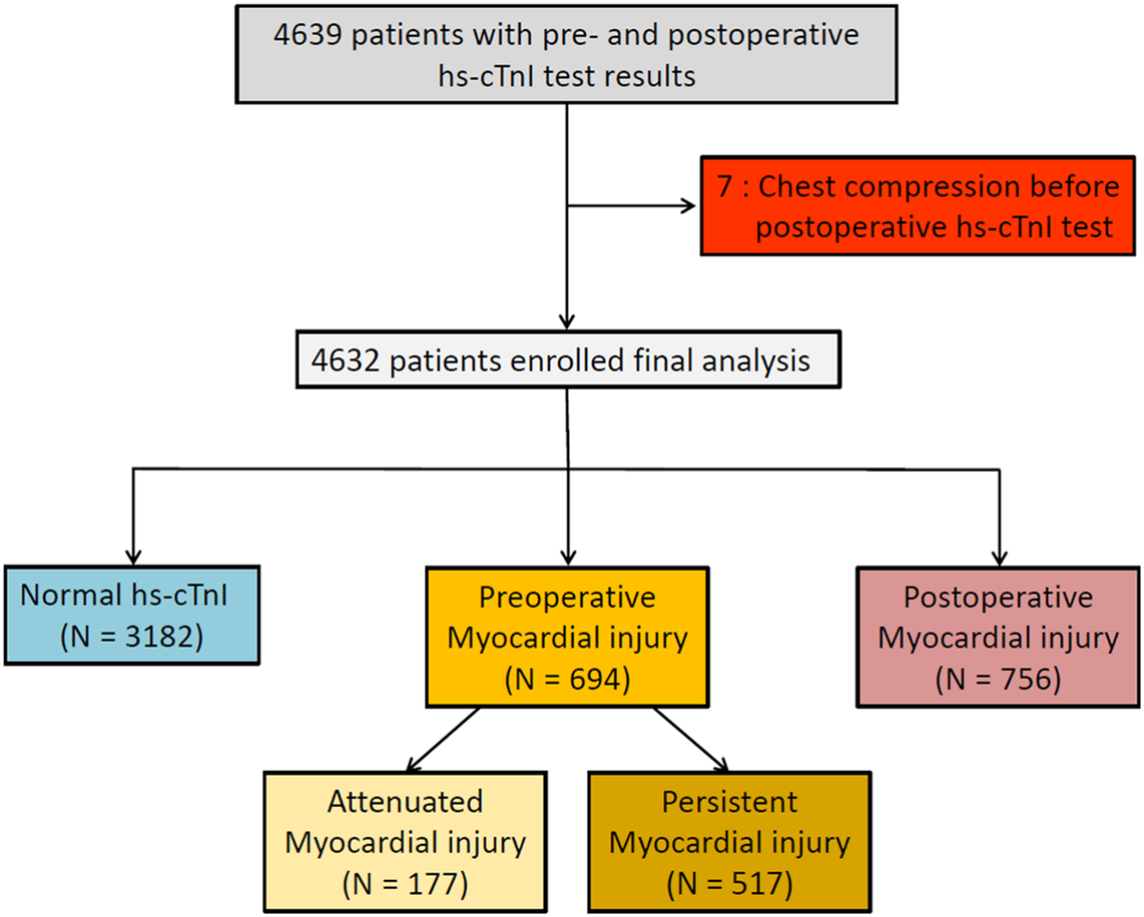
The flowchart of the patients. Hs-cTnI, high sensitivity-cardiac troponin I.

The flowchart of the patients is presented in Fig. 1. Of the enrolled 4632 patients, 694 (4.8%) patients with preoperative hs-cTn elevation were stratified into the preoperative myocardial group. After stratifying 756 (4.1%) patients with postoperative hs-cTn elevation into the postoperative myocardial group, the remaining 3182 (91.1%) patients with normal hs-cTn level at pre- and postoperative measurements were stratified into the normal group. The patients in the preoperative myocardial group were further divided into two groups according to postoperative myocardial injury, and 177 patients were in the attenuated (25.5%) group and 517 patients in the persistent (74.5%) myocardial groups. So, the total number of patients with elevated hs-cTn level in the postoperative period was 1273 (27.5% of the entire population).

The baseline characteristics of the three groups are summarized in Table 1, and the types of surgery were described in Supplementary Table S1. Regarding the normal group as a reference group, the preoperative myocardial injury group showed higher rate of diabetes, history of coronary revascularization, heart failure, arrhythmia, stroke, chronic kidney disease, chronic lung disease, and infectious state than the normal group. In addition, the preoperative group showed increased use of preoperative beta-blocker and decreased use of statin. The postoperative myocardial injury group showed higher rate of diabetes, history of coronary revascularization, heart failure, valve disease, chronic kidney disease, and aortic disease than the normal group. Preoperative beta-blocker therapy showed also higher incidence in the preoperative myocardial injury group. In the blood test, the pre- and post-operative myocardial injury group showed decreased hemoglobin and increased creatinine and liver enzyme levels than the normal group. Regarding the operative variables, the postoperative myocardial injury group showed higher incidence of high-risk operation than the other two groups. The rate of emergent operation, intraoperative inotropic and colloid use were higher in both myocardial injury groups compared to the normal group. The postoperative myocardial injury group showed the longest operative duration. After an inverse probability weighting adjustment, standardized mean differences less than 10% suggested a successful balance between the three groups (Table 1).

**Table 1:** Baseline characteristics of the study population.

	Entire population	Normal (*N* = 3182)	Preoperative myocardial injury (*N* = 694)	Postoperative myocardial injury (*N* = 756)	*p* value	SMD	IPW	
							*p* value	SMD
**Demographic variables**								
Male sex	2852 (61.6)	1957 (61.5)	411 (59.2)	484 (64)	0.17	6.6	0.27	7.6
Age, years	65.6 (± 13.9)	65.1 (± 12.8)	66.7 (± 14.1)	66.6 (± 13.2)		8.1	0.08	6.4
BMI	23.6 (± 3.83)	23.9 (± 3.8)	22.8 (± 3.9)	23.3 (± 4.0)		19.5	0.51	4
**Previous history**								
Hypertension	2464 (53.2)	1665 (52.4)	368 (53.0)	430 (56.9)	0.08	6.1	0.62	4
Diabetes	1314 (28.4)	836 (26.3)	246 (35.4)	232 (30.7)	< 0.001	13.3	0.76	2.5
Current smoking	572 (12.4)	410 (12.9)	76 (11.0)	86 (11.4)	0.25	4	0.94	1.2
Previous myocardial infarction	279 (6.0)	156 (4.9)	78 (11.2)	45 (6.0)	< 0.001		0.78	2.3
Coronary revascularization	714 (15.4)	417 (13.1)	144 (20.7)	153 (20.2)	< 0.001	13.7	0.36	4.3
Heart failure	137 (3.0)	53 (1.7)	59 (8.5)	25 (3.3)	< 0.001	21.4	0.06	7.7
Arrhythmia	486 (10.5)	285 (9.0)	112 (16.1)	89 (11.8)	< 0.001	14.6	0.33	4.1
Valve disease	139 (3.0)	77 (2.4)	27 (3.9)	35 (4.6)	0.002	8	0.55	3.1
Stroke	666 (14.4)	410 (12.9)	127 (18.3)	129 (17.1)	< 0.001	10	0.81	1.8
Chronic kidney disease	478 (10.3)	185 (5.8)	164 (23.6)	129 (17.1)	< 0.001	34.7	0.34	4.7
Aortic disease	289 (6.2)	167 (5.2)	38 (5.5)	84 (11.1)	< 0.001	14.4	0.5	3.7
PAD	434 (9.4)	294 (9.2)	75 (10.8)	65 (8.6)	0.32	5	0.46	5.7
PTE/DVT	91 (2.0)	64 (2.0)	21 (3.0)	6 (0.8)	0.01	11.1	0.99	0.1
Cancer	1017 (22.0)	696 (21.9)	163 (23.5)	158 (20.9)	0.48	4.2	0.29	6.8
Chronic lung disease	597 (12.9)	380 (11.9)	119 (17.1)	98 (13)	0.001	9.9	0.26	6.2
Infectious disease	2170 (46.9)	1286 (40.4)	521 (75.1)	363 (48.0)	< 0.001	49.4	0.45	4.9
**Preoperative medication**								
Beta blocker	897 (19.4)	563 (17.7)	147 (21.2)	187 (24.7)	< 0.001	11.5	0.42	4.7
RAAS inhibitor	1281 (27.7)	891 (28.0)	180 (25.9)	210 (27.8)	0.54	3.1	0.52	5.4
Statin	1214 (26.2)	853 (26.8)	143 (20.6)	218 (28.8)	0.001	12.8	0.82	2.3
Antiplatelet	1469 (31.7)	975 (30.6)	222 (32.0)	272 (36.0)	0.02	7.6	0.84	1.5
**Preoperative blood test**								
Hemoglobin	11.9 (± 2.2)	12.2 (± 2.1)	10.7 (± 2.1)	11.6 (± 2.2)		46.6	0.67	3.4
Creatinine	1.27 (± 1.59)	1.03 (± 1.19)	2.05 (± 2.46)	1.53 (± 1.79)		36.7	0.6	2.8
AST	41.1 (± 144.5)	30 (± 56)	87 (± 291)	44 (± 187)		18	0.18	4.5
ALT	35.9 (± 122.4)	3 (± 50)	62 (± 200)	39 (± 209)		13.1	0.14	6.2
**Operative variables**								
ESC/ESA Risk					< 0.001	32	0.72	6.5
High	733 (15.8)	460 (14.5)	74 (10.7)	199 (26.3)				
Intermediate	3409 (73.6)	2396 (75.3)	510 (73.5)	503 (66.5)				
Low	490 (10.6)	326 (10.2)	110 (15.9)	54 (7.1)				
Operative duration, hours	3.26 (± 2.61)	3.12 (± 2.25)	2.94 (± 2.99)	4.15 (± 3.38)		26.9	0.96	0.8
General anesthesia	4197 (90.6)	2892 (90.9)	620 (89.3)	685 (90.6)	0.45	3.5	0.15	8.2
Emergent operation	1190 (25.7)	650 (20.4)	314 (45.2)	226 (29.9)	< 0.001	36.3	0.25	5.2
Inotropic use	1315 (28.4)	637 (20.0)	281 (40.5)	397 (52.5)	< 0.001	47.3	0.63	3.3
Colloid use	2324 (50.2)	1488 (46.8)	360 (51.9)	476 (63.0)	< 0.001	21.9	0.43	4.8
RBC transfusion, pints	0.78 (± 2.1)	0.7 (± 0.6)	0.9 (± 1.0)	1.0 (± 1.0)		24.2	0.97	0.8

Values are n (%) or mean (± SD).

*SMD* standardized mean difference, *BMI* body mass index, *PAOD* peripheral artery disease, *PTE/DVT* pulmonary thromboembolism/deep vein thrombosis, *RAAS* renin–angiotensin–aldosterone system, *AST* aspartate aminotransferase, *ALT* alanine aminotransferase.

**Figure 2:**
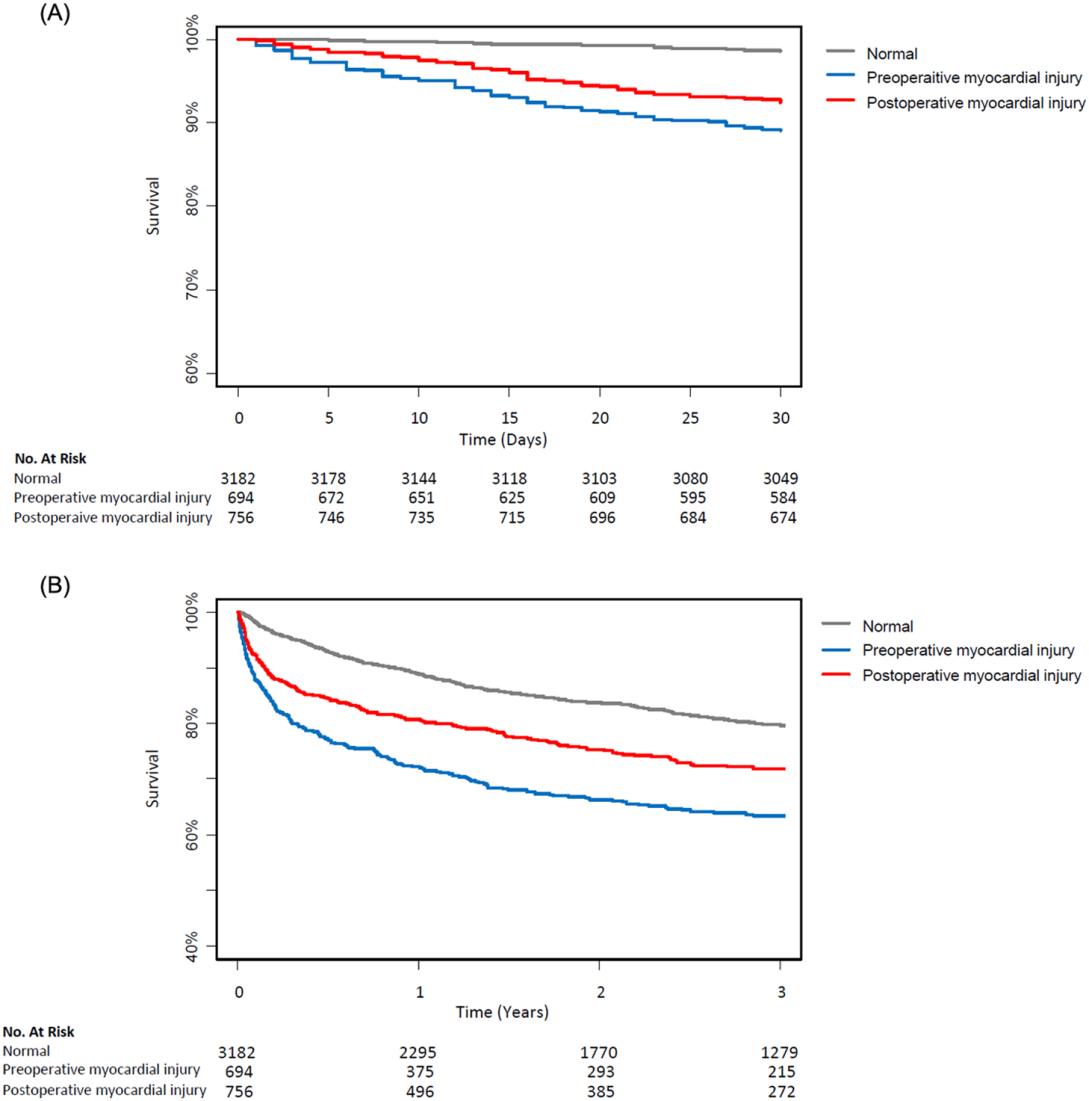
Kaplan–Meier curves for the normal group (grey line), preoperative myocardial injury group (blue line) and postoperative myocardial injury group (red line). Curves for (**A**) 30-day mortality, (**B**) mortality during follow-up.

Clinical outcomes were compared pairwise. The 30-day mortalities were 1.4% in the normal group, 10.7% in the preoperative myocardial injury group, and 7.4% in the postoperative myocardial injury group, respectively. In univariate Cox regression analysis, both pre-and postoperative myocardial injury was significantly associated with increased 30-day mortality (HR 8.31; 95% CI 5.72–12.07; *p* < 0.001; HR 2.36; 95% CI 1.94–2.88; *p* < 0.001, respectively) (Table 2, Fig. 2). After inverse probability weighting adjustment, the result showed consistent 30-day mortality differences (HR 3.12; 95% CI 1.62–6.01; *p* = 0.001; HR 4.49; 95% CI 4.49; 95% CI 2.34–8.60; *p* < 0.001), and it was not significantly different between the pre-and postoperative myocardial injury groups (HR 1.44; 95% CI 0.79–2.64; *p* = 0.45) (Table 2). Acute kidney injury was increased in both myocardial injury group (OR 2.13; 95% CI 1.43–3.19; *p* < 0.001; OR 3.11; 95% CI 2.30–4.21; *p* < 0.001, respectively) (Table 2). For other secondary outcomes, the median follow-up durations were 34.3 (± 27.8) months in the normal, 26.2 (± 27.8) months in the preoperative, and 30.3 (± 26.7) months in the postoperative myocardial injury groups, and the incidences were compared in Table 2.

**Table 2:** Clinical outcomes.

	n (%)	Univariate analysis		IPW analysis	
		Unadjusted HR (95% CI)	*p* value	Adjusted HR (95% CI)	*p* value
**30-day mortality**					
Normal	44 (1.4)	1		1	
Preoperative myocardial injury	74 (10.7)	8.31 (5.72–12.07)	< 0.001	3.12 (1.62–6.01)	0.001
Postoperative myocardial injury	56 (7.4)	2.36 (1.94–2.88)	< 0.001	4.49 (2.34–8.60)	< 0.001
Pre- vs. postoperative myocardial injury		0.67 (0.47–0.95)	0.02	1.44 (0.79–2.64)	0.45
**In-hospital death**					
Normal	65 (2.0)	1		1	
Preoperative myocardial injury	97 (14)	3.33 (2.42–4.60)	< 0.001	2.87 (0.95–3.68)	0.01
Postoperative myocardial injury	71 (9.4)	1.62 (1.37–1.92)	< 0.001	2.32 (1.16–4.65)	0.001
Pre- vs. postoperative myocardial injury		0.80 (0.59–1.09)	0.16	1.24 (0.68–2.26)	0.34
**Overall cardiovascular death**					
Normal	201 (6.3)	1		1	
Preoperative myocardial injury	71 (10.2)	2.07 (1.58–2.72)	< 0.001	1.30 (0.89–1.89)	0.18
Postoperative myocardial injury	71 (9.4)	1.29 (1.13–1.48)	< 0.001	1.61 (1.16–2.23)	0.004
Pre- vs. postoperative myocardial injury		0.81 (0.58–1.13)	0.21	1.21 (0.70–2.10)	> 0.99
**Overall all-cause death**					
Normal	596 (18.7)	1		1	
Preoperative myocardial injury	233 (33.6)	2.24 (1.92–2.60)	< 0.001	1.37 (1.08–1.74)	0.009
Postoperative myocardial injury	218 (28.8)	1.30 (1.20–1.41)	< 0.001	1.51 (1.24–1.85)	< 0.001
Pre- vs. postoperative myocardial injury		0.77 (0.64–0.93)	0.006	1.10 (0.79–1.54)	> 0.99

	n (%)	Univariate analysis		IPW analysis	
		Unadjusted OR (95% CI)	*p* value	Adjusted OR (95% CI)	*p* value
**Type 1 myocardial infarction**					
Normal	10 (0.3)	1		1	
Preoperative myocardial injury	8 (1.2)	3.70 (1.41–9.41)	0.006	2.07 (0.59–7.25)	0.49
Postoperative myocardial injury	16 (2.1)	2.62 (1.77–3.96)	< 0.001	7.12 (2.54–19.99)	< 0.001
Pre- vs. postoperative myocardial injury		1.85 (0.81–4.60)	0.16	3.44 (1.02–11.56)	0.04
**Coronary revascularization**					
Normal	6 (0.2)	1		1	
Preoperative myocardial injury	3 (0.4)	2.30 (0.48–8.73)	0.24	2.08 (0.33–12.99)	0.99
Postoperative myocardial injury	8 (1.1)	2.38 (1.40–4.15)	0.001	6.29 (1.58–24.98)	0.004
Pre- vs. postoperative myocardial injury		2.46 (0.71–11.28)	0.18	3.03 (0.49–18.83)	0.44
**Atrial fibrillation**					
Normal	67 (2.1)	1		1	
Preoperative myocardial injury	52 (7.5)	3.77 (2.59–5.45)	< 0.001	3.34(1.76–6.35)	< 0.001
Postoperative myocardial injury	47 (6.2)	1.76 (1.45–2.12)	< 0.001	2.61 (1.54–4.43)	< 0.001
Pre- vs. postoperative myocardial injury		0.82 (0.54–1.23)	0.34	0.78 (0.39–1.57)	0.99
**Heart failure**					
Normal	3 (0.1)	1		1	
Preoperative myocardial injury	8 (1.2)	12.36 (3.56–56.52)	< 0.001	15.22 (2.49–93.16)	< 0.001
Postoperative myocardial injury	4 (0.5)	2.37 (1.11–5.35)	0.02	4.15 (0.62–28.02)	0.22
Pre- vs. postoperative myocardial injury		0.46 (0.12–1.45)	0.2	0.27 (0.05–1.58)	0.99
**AKI, any**					
Normal	205 (6.4)	1		1	
Preoperative myocardial injury	172 (24.8)	4.79 (3.83–5.98)	< 0.001	2.13 (1.43–3.19)	< 0.001
Postoperative myocardial injury	219 (29.0)	2.43 (2.19–2.71)	< 0.001	3.11 (2.30–4.21)	< 0.001
Pre- vs. postoperative myocardial injury		1.24 (0.98–1.56)	0.07	1.46 (0.96–2.22)	0.1

*AKI* acute kidney injury.

**Figure 3:**
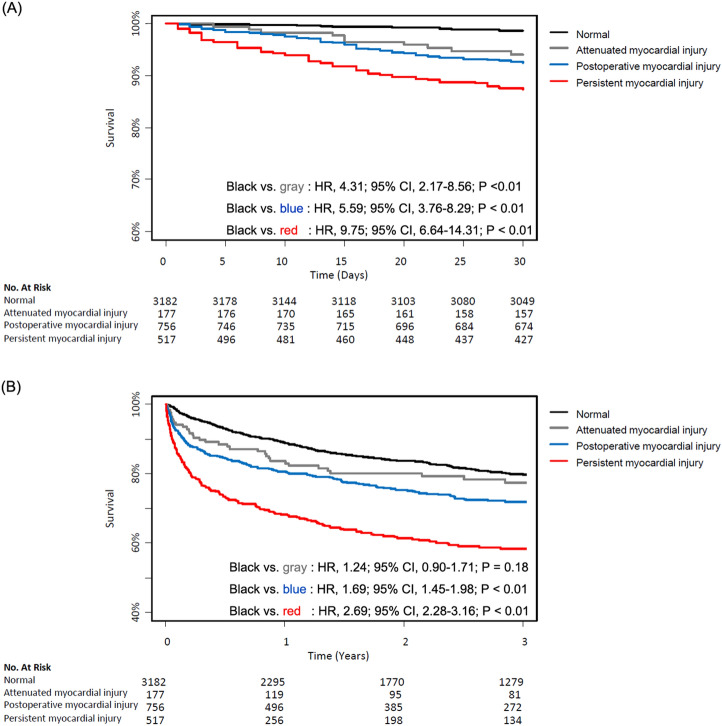
Kaplan–Meier curves for the normal group (black line), attenuated myocardial injury group (grey line), postoperative myocardial injury group (blue line) and persistent myocardial injury group (red line). Curves for (**A**) 30-day mortality, (**B**) mortality during follow-up.

Additionally, the patients with attenuation of preoperative myocardial injury were compared to those with persistent myocardial injury (Supplementary Table S2). 30-day mortality was significantly lower for the attenuated myocardial injury group (5.6% vs. 12.4%; HR 2.23; 95% CI 1.17–4.44; *p* = 0.02) (Table 3). The survival curves are shown in Fig. 3.

**Table 3:** Clinical outcomes of preoperative myocardial injury group.

	Attenuated myocardial injury (*N* = 177)	Persistent myocardial injury (*N* = 517)	Unadjusted HR (95% CI)	*p* value
30-day mortality	10 (5.6)	64 (12.4)	2.23 (1.17–4.44)	0.02
In-hospital death	11 (6.2)	86 (16.6)	2.10 (1.12–3.94)	0.02
Overall cardiovascular death	14 (7.9)	57 (11.0)	1.82 (1.01–3.26)	0.05
Overall all-cause death	40 (22.6)	193 (37.3)	2.03 (1.44–2.86)	< 0.001

	Attenuated myocardial injury (*N* = 177)	Persistent myocardial injury (*N* = 517)	Unadjusted OR (95% CI)	*p* value
Type 1 myocardial infarction	1 (0.6)	7 (1.4)	2.42 (0.43–45.3)	0.41
Coronary revascularization	0	3 (0.6)		
Atrial fibrillation	8 (4.5)	44 (8.5)	1.97 (0.96–4.58)	0.09
Heart failure	0	8 (1.5)		
AKI, any	21 (11.9)	151 (29.2)	3.06 (1.91–5.14)	< 0.001

*AKI* acute kidney injury.

## Discussion

Our study showed that both pre- and postoperative myocardial injuries were associated with increased 30-day mortality after non-cardiac surgery and the mortality rates were similar between the patients with pre- and postoperative myocardial injuries based on the 4th universal definition of myocardial infarction. In addition, attenuation of preoperative myocardial injury appeared to be related with the improved postoperative outcomes compared with persistent myocardial injury.

In the fourth universal definition of myocardial infarction, type 2 myocardial infarction was classified into acute myocardial infarction and myocardial injury, which was previously integrated in the previous definition^14^. Myocardial injury is defined as a sole elevation of cTn value above the 99th percentile upper reference limit without ischemic symptom, and the definition of myocardial infarction requires a clinical evidence of acute myocardial ischemia in addition to myocardial injury^14^. Although Type 2 myocardial infarction and myocardial injury are frequently encountered in surgical patients^14,15^, discrimination between type 2 myocardial infarction and myocardial injury still remains challenging during the perioperative period, because clinical evidence of acute myocardial ischemia such as chest pain or dyspnea may be masked due to anaesthesia or analgesia.

Myocardial injury after non-cardiac surgery, which uses a cut-off point upper than 99th percentile as an upper-reference value of cTn^14^, occurs in almost 20% of the patients who underwent non-cardiac surgery^16^ and has been recently accepted as a strong predictor of early postoperative mortalities^6,15^. Mortality within 30 days in patients with myocardial injury after non-cardiac surgery has been reported as around 10%, which represents a more than 5-fold increase from the background risk^17^. Considering that 30-day mortalities were 1.4% in the normal group, 10.7% in the preoperative myocardial injury group, and 7.4% in the postoperative myocardial injury group in the present study, our results are correspondent with previous data. However, the incidences of pre- and postoperative myocardial injury were 4.8% and 4.1% in our study, respectively, which were lower than the previously reported incidences. We thought that separation of pre- and postoperative myocardial injuries and difference in surgical characteristics might have made this difference. Another issue to consider is that ischemic feature such as electrocardiographic change or cardiac symptom was not considered in this study. Due to the retrospective nature, perioperative cardiac monitoring was not controlled during the study period, and chest pain in the perioperative period is likely to be masked by sedatives or analgesics. So, the incidence and effect of perioperative myocardial injury with ischemic feature need further studies.

Preoperative cardiac troponin elevation, by reflecting underlying condition of the patient has also shown a significant association with postoperative mortality^9–11^ In some studies focused on postoperative cTn elevation, preoperative cTn elevation was considered as a chronic elevation and excluded from the analysis^6,16,18^, because serum cTn elevation persist for at least 5 days^19^, and patients with preoperative myocardial injury can theoretically be diagnosed as those with postoperative myocardial injury regardless of developing intra- or postoperative myocardial injury. In this study, we compared the effect of pre- and postoperative myocardial injury on 30-day mortality simultaneously and showed that 30-day mortality rates were similar between the two groups. Therefore, our result seems to add an evidence for the need of close monitoring for patients with preoperative myocardial injury as well as postoperative myocardial injury in patients undergoing non-cardiac surgery.

For further analysis, the preoperative myocardial injury group was divided according to postoperative normalization of hs-cTn I, and the mortality was compared. Interestingly, an attenuated myocardial injury after surgery was associated with lower 30-day mortality compared to persistent myocardial injury. This finding suggests that intraoperative and early postoperative intervention for preoperatively elevated hs-cTn could be related to postoperative outcomes. Although a modality to normalize hs-cTn has not been established, an appropriate perioperative managements according to current guidelines still seems to be important in patients with preoperative myocardia injury, because preoperative, intraoperative, and postoperative risk factors are all related to the development of myocardial injury after non-cardiac surgery^20^. However, further studies with larger registry should be needed to confirm our findings.

Our results should be appraised in the light of the following limitations. First, the present study is a single-center and retrospective analysis. Therefore, despite an adjustment with the inverse probability weighting, the results could be affected by the confounding factors since unmeasured variables were not able to be corrected even after an adjustment. Second, in our institution, the measurement of hs-cTn level is not a routine perioperative laboratory examination in non-cardiac surgery, and the time point and numbers of perioperative hs-cTn measurements were not controlled. Considering that this study selected the patients who needed hs-cTn measurements in both pre- and postoperative periods, the incidence of hs-cTn elevation was higher than previously reported studies. So, our results in high-risk patients might have a chance to be exaggerated. Lastly, detailed perioperative cardiac evaluation such as left ventricular ejection fraction on echocardiography or coronary artery angiograms, was not available in all patients, and the presence of ischemic feature was not considered in the analysis.

## Methods

This retrospective analysis included adult patients who had undergone non-cardiac surgery at Samsung Medical Center (Seoul, Korea). The study protocol was approved by the Institutional Review Board of Samsung Medical Center (IRB No. 2017-10-109-003) and registered in Clinical Research Information Service (KCT0004348). This study was also conducted in accordance with the principles of the Declaration of Helsinki. Since the present study only used the routinely gathered patient data and had no risk of enrolled patients, the need for individual informed consent was waived by our Institutional Review Board.

### Patients and management

We included all adult non-cardiac surgical patients who has the hs-cTn results within 48 h before and after surgery at Samsung Medical Center between January, 2010 and December, 2016. Hs-cTn levels within 48 h both before and after surgery were measured in 4639 (2.2%) adult patients from a total of 210,322 adult patients who underwent non-cardiac surgery in our institution during the same period. After excluding 7 patients in whom cardiopulmonary resuscitation was performed before postoperative hs-cTn measurement, 4632 patients were enrolled into the analysis. According to the time point of peak hs-cTn elevation above the 99th percentile of upper reference limit, the patients were stratified into either of the following three groups; the normal (*n* = 3182), preoperative myocardial injury (*n* = 694), and postoperative myocardial injury (*n* = 756). The numbers of hs-cTn measurement in the postoperative period were 1 (interquartile range 1–3) in the normal, 1 (interquartile range 1–3) in the preoperative, and 2 (interquartile range 1–4) in the postoperative myocardial injury groups. Time intervals from the end of surgery to peak level of hs-cTn were 17.1 (interquartile range 12.6–32.0), 17.9 (interquartile range 12.1–36.9), and 17.9 (interquartile range 12.1–36.9) hours in the normal, preoperative, and postoperative myocardial injury groups, respectively.

Myocardial injury was defined as a sole elevation of cTn value above the 99th percentile of upper reference limit based on the 4th universal definition of myocardial infarction^14^. The patients in the preoperative myocardial injury group were further divided into the attenuated or persistent myocardial injury group according to the normalization postoperative hs-cTn level below 99th percentile of upper reference limit (Fig. 1).

Perioperative evaluation and management including hs-cTn measurement were performed according to our institutional protocol based on the current non-cardiac surgery guideline from European Society of Cardiology and the European Society of Anaesthesiology^25^. Following the institutional protocol, hs-cTn I was measured for moderate- or high-risk surgery or in patients with at least one of the major cardiovascular risk factors such as a history of ischemic heart disease, heart failure, stroke including transient ischemic attack, diabetes mellitus on insulin therapy, or chronic kidney disease. It was also measured in patients with minor risk factors at the discretion of the attending clinician, considering old age or recently suspected symptoms of ischemic disease. An automated analyzer (Advia Centaur XP, Siemens Healthcare Diagnostics, Erlangen, Germany) was used for measuring hs-cTn level. Lowest limit of detection was 6 ng/L, and normal upper limit was 40 ng/L according to the 99th percentile reference^21^.

### Data collection and extraction

Data were obtained from a paperless electronic medical record system of Samsung Medical Center. Initially, the patients with both pre- and postoperative hs-cTn measurement were identified with the aid of our Institutional Medical Information Department. Following the initial data collection, the “Clinical Data Warehouse Darwin-C” program which was designed for searching and retrieving the deidentified medical records was used for the further data extraction. In this system, death of patients was consistently updated from the national database. An investigator (J.J. Min) who was blinded to the serum hs-cTn level organized baseline patients’ characteristics and postoperative clinical outcomes except death were collected through manual review of each patient’s medical records by the independent investigators (H. Cho and K.Y. Hong) who were blinded to the serum hs-cTn level and baseline characteristics.

### Study outcomes and definitions

Definitions of clinical outcomes were based on a report on cardiovascular events in clinical trials by American College of Cardiology Foundation/American Heart Association task force^22^. The primary outcome was 30-day mortality. Secondary outcomes included in-hospital mortality, overall mortality, cardiovascular mortality, and type 1 myocardial infarction, coronary revascularization, stroke, newly developed atrial fibrillation, newly developed heart failure, and postoperative acute kidney injury during hospital stay. Type 1 myocardial infarction was defined as an angiographically proven myocardial infarction according to the 4th universal definition^14^. Heart failure was defined when the patient exhibits new or worsening symptoms of heart failure on presentation, has objective evidence of new or worsening heart failure, and receives initiation or intensification of treatment specifically for heart failure. Postoperative acute kidney injury was defined as either an increase in serum creatinine greater than or equal to 0.3 mg/dL within postoperative 48 h or an increase to greater than or equal to 1.5 times baseline within seven days according to the Kidney Disease Improving Global Outcomes criteria^23^.

### Statistical analysis

We used analysis of variance tests or Kruskal–Wallis tests to compare differences in baseline characteristics, as applicable, and presented as mean ± standard deviation (SD) or median with interquartile range for continuous variables. Kaplan–Meier estimates were used to construct survival curves and compared with the unadjusted log-rank test. Cox regression was used to evaluate 30-day, in-hospital, overall and cardiovascular mortalities, and logistic regression was used to compare other outcomes during hospital stay. To further reduce selection bias and maximize the study power while maintaining a balance in confounding factors between the three groups, we conducted rigorous adjustment for differences in baseline characteristics of patients using the weighted Cox proportional-hazards regression models with the inverse probability weightingy^24^. The inverse probability weights were defined as the reciprocal of propensity scores that were calculated by the generalized boosted model for estimating the average effects and the probability of being in each of the three study groups. Variables retained in this adjustment included dermographic variables, previous medical history, preoeprative medication, results of preoperative blood tests, and operative variables. After an adjustment, standardized mean difference less than 10% was deemed as a good balance between the groups. An inverse probability weighting adjusted cox regression was used to evaluate all-cause, 30-day and in-hospital mortality. We adopted a post hoc Bonferroni correction, which allowed for the primary outcome to be tested at an alpha level of 0.0167 (0.05 ÷ 3). The reduction in the risk of outcome was compared using either Cox or logistic regression model, as applicable. Adjusted hazard ratio (HR) or odds ratio (OR) with 95% confidence interval (CI) was reported for immediate clinical outcomes. Statistical analyses were performed with SAS version 9.4 (SAS Institute, Cary, NC) and R 3.5.3 (Vienna, Austria; http://www.R-project.org/). All tests were two-tailed and *p* < 0.05 was considered statistically significant.

### Conference presentation

This study was partly presented in the 96th Annual Scientific Meeting of the Korean Society of Anesthesiologists (KoreAnesthesia 2019), Oct 31-Nov 2, 2019, Incheon, Korea.

## Conclusion

Our study showed that preoperative myocardial injury significantly increased 30-day mortality in the patients undergoing non-cardiac surgery to a similar degree of postoperative myocardial injury. In addition, it might be related with the improvement of 30-day mortality after non-cardiac surgery to attenuate the preoperative cTn elevation.

## Supplementary information

Supplementary Information.

## Abbreviations

cTn: Cardiac troponin
Hs-cTn: High-sensitivity-cardiac troponin
SD: Standard deviation
IQR: Interquartile range
HR: Hazard ratio
OR: Odds ratio
