# Supplementary material for: Comparison of pre- and postoperative myocardial injuries on mortality after non-cardiac surgery: a retrospective analysis using an inverse probability weighting adjustment
Source: Sci Rep. 2020 Dec 3;10:21050. doi: 10.1038/s41598-020-78023-9 (PMC7713127; doi:10.1038/s41598-020-78023-9)
Supplement: Supplementary file 1 — Supplementary Information. [file 41598_2020_78023_MOESM1_ESM.docx]

**Comparison between the Effects of Pre- and Postoperative Myocardial Injuries on 30-Day Mortality After Non-Cardiac Surgery: A Retrospective Analysis Using an Inverse Probability Weighting Adjustment**

Seung-Hwa Lee, MD^1^; Jungchan Park, MD^2^; Jong-Hwan Lee, MD, PhD^2^; Jeong Jin Min, MD, PhD^2^; Kwan Young Hong, MD^2^; Hyojin Cho, MD^2^; Keumhee Carriere, PhD^3,4^; Joonghyun Ahn, PhD^4^

^1^Division of Cardiology, Department of Medicine, Heart Vascular Stroke Institute, Samsung Medical Center, Sungkyunkwan University School of Medicine, Seoul, Korea.

^2^Department of Anesthesiology and Pain Medicine, Samsung Medical Center, Sungkyunkwan University School of Medicine, Seoul, Korea.

^3^Department of Mathematical and Statistical Sciences, University of Alberta, Edmonton, AB, Canada. ^4^Statistics and Data Center, Samsung Medical Center, Sungkyunkwan University School of Medicine, Seoul, Korea.

Address correspondence to Dr. Jong-Hwan Lee: Department of Anesthesiology and Pain Medicine, Samsung Medical Center, Sungkyunkwan University School of Medicine, 81 Irwon-ro, Gangnam-gu, Seoul 06351, Korea. jonghwanlee75@gmail.com.

**Table S1.** Operation type

|  | **Normal**  **(*N* = 3182)** | **Preoperative myocardial injury**  **(*N* = 694)** | **Postoperative myocardial injury**  **(*N* = 756)** |
| --- | --- | --- | --- |
| Vascular | 829 (26.1) | 105 (15.1) | 220 (29.1) |
| Abdominal | 944 (29.7) | 267 (38.5) | 260 (34.4) |
| Orthopedic | 464 (14.6) | 168 (24.2) | 131 (17.3) |
| Thoracic | 720 (22.6) | 73 (10.5) | 90 (11.9) |
| Neurogenic | 109 (3.4) | 44 (6.3) | 18 (2.4) |
| ENT, EYE | 116 (3.6) | 37 (5.3) | 37 (4.9) |

**Table S2.** Baseline characteristics of the preoperative myocardial injury group

|  | **Attenuated myocardial injury**  **(*N* = 177)** | **Persistent myocardial injury (*N* = 517)** | ***p* value** |
| --- | --- | --- | --- |
|  |  |  |  |
| **Demographic variables** |  |  |  |
| Male sex | 103 (58.2) | 308 (59.6) | 0.82 |
| Age, years | 64.7 (±14.9) | 67.4 (±13.8) | 0.03 |
| BMI | 23.4 (±3.76) | 22.6 (±3.93) | 0.02 |
| **Previous history** |  |  |  |
| Hypertension | 93 (52.5) | 275 (53.2) | 0.95 |
| Diabetes | 49 (27.7) | 197 (38.1) | 0.02 |
| Current smoking | 28 (15.8) | 48 (9.3) | 0.02 |
| Old myocardial infarction | 15 (8.5) | 63 (12.2) | 0.23 |
| Coronary revascularization | 30 (16. | 153 (20.2) | 0.86 |
| Heart failure | 30 (16.9) | 114 (22.1) | 0.18 |
| Arrythmia | 28 (15.8) | 84 (16.2) | 0.99 |
| Valve disease | 4 (2.3) | 23 (4.4) | 0.28 |
| Stroke | 26 (14.7) | 101 (19.5) | 0.19 |
| Chronic kidney disease | 20 (11.3) | 144 (27.9) | <0.001 |
| Aortic disease | 7 (4.0) | 31 (6.0) | 0.4 |
| PAD | 17 (9.6) | 58 (11.2) | 0.65 |
| PTE/DVT | 7 (4.0) | 14 (2.7) | 0.56 |
| Cancer | 49 (27.7) | 114 (22.1) | 0.16 |
| Chronic lung disease | 27 (15.3) | 92 (17.8) | 0.51 |
| Infectious state | 135 (76.3) | 386 (74.7) | 0.74 |
| **Preoperative medication** | |  |  |
| Beta blocker | 34 (19.2) | 113 (21.9) | 0.52 |
| RAAS inhibitor | 44 (24.9) | 136 (26.3) | 0.78 |
| Statin | 29 (16.4) | 114 (22.1) | 0.13 |
| Antiplatelet | 45 (25.4) | 177 (34.2) | 3.8 |
| **Preoperative blood test** |  |  |  |
| Hemoglobin | 10.8 (±2.1) | 10.7 (±2.1) | 0.58 |
| Creatinine | 1.35 (±1.94) | 2.29 (±2.57) | <0.001 |
| AST | 43 (±81) | 102 (±333) | 0.02 |
| ALT | 36 (±67) | 71 (±228) | 0.04 |
| **Operative variables** |  |  |  |
| Risk |  |  | 0.2 |
| High | 22 (12.4) | 52 (10.1) |  |
| Intermediate | 121 (68.4) | 389 (75.2) |  |
| Low | 34 (19.2) | 76 (14.7) |  |
| Operative duration, hours | 3.08 (±3.37) | 2.89 (±2.85) | <0.001 |
| General anesthesia | 167 (94.4) | 453 (87.6) | 0.02 |
| Emergent operation | 71 (40.1) | 243 (47.0) | 0.13 |
| Inotropic use | 47 (26.6) | 234 (45.3) | <0.001 |
| Colloid use | 79 (44.6) | 281 (54.4) | 0.03 |
| RBC transfusion, pints | 0.71 (±0.71) | 1.01 (±1.04) | <0.001 |

Values are n (%) or mean (±SD)

SMD: standardized mean difference; BMI: body mass index; PAOD: peripheral artery disease; PTE/DVT: pulmonary thromboembolism/deep vein thrombosis; RAAS: renin-angiotensin-aldosterone system; AST: aspartate aminotransferase; ALT: alanine aminotransferase
